# MEMS Gyroscope Bias Drift Self-Calibration Based on Noise-Suppressed Mode Reversal

**DOI:** 10.3390/mi10120823

**Published:** 2019-11-27

**Authors:** Haoyu Gu, Baolin Zhao, Hao Zhou, Xianxue Liu, Wei Su

**Affiliations:** Institute of Electronic Engineering, China Academy of Engineering Physics, Mianyang 621999, China; guhaoyu19901015@163.com (H.G.); chinamems@163.com (H.Z.); liuxx19901015@163.com (X.L.); suwei19901015@163.com (W.S.)

**Keywords:** MEMS gyroscope, Bias drift self-calibration, Mode reversal, Improved CEEMDAN

## Abstract

This paper presents a bias drift self-calibration method for micro-electromechanical systems (MEMS) gyroscopes based on noise-suppressed mode reversal without the modeling of bias drift signal. At first, the bias drift cancellation is accomplished by periodic switching between operation mode of two collinear gyroscopes and subtracting the bias error which is estimated by the rate outputs from a consecutive period interval; then a novel filtering algorithm based on improved complete ensemble empirical mode decomposition (improved complete ensemble empirical mode decomposition with adaptive noise—CEEMDAN) is applied to eliminate the noise in the calibrated signal. A set of intrinsic mode functions (IMFs) is obtained by the decomposition of the calibrated signal using improved CEEMDAN method, and the threshold denoising method is utilized; finally, the de-noised IMFs are reconstructed into the desired signal. To verify the proposed method, the hardware circuit with an embedded field-programmable gate array (FPGA) was implemented and applied in bias drift calibration for the two MEMS gyroscopes manufactured in our laboratory. The experimental results indicate that the proposed method is feasible, and it achieved a better performance than the typical mode reversal. The bias instability of the two gyroscopes decreased from 0.0066°/s and 0.0055°/s to 0.0011°/s; and, benefiting from the threshold denoising based on improved CEEMDAN, the angle random walks decreased from 1.18 $\times 10^{-4}{}^{\circ}/s^{1/2}$ and 2.04 $\times 10^{-4}{}^{\circ}/s^{1/2}$ to 2.19 $\times 10^{-5}{}^{\circ}/s^{1/2}$, respectively.

## 1. Introduction

Micro-electromechanical system (MEMS) gyroscopes are developing rapidly due to their extensive applications in the field of consumer electronics and in the military, such as game stations, cameras, vehicle control systems and weapon guidance. Compared with traditional gyroscopes, the MEMS gyroscopes have the advantages of low costs, low power consumption and small size [1].

The major problem for MEMS gyroscopes is to reach the requirement of inertial grade which means a greatly improvement in the performance of bias stability. Therefore, a large number of studies have been conducted, including on the vibrating poly-silicon ring gyroscope, the tuning fork SOI (silicon on insulator) gyroscope with mode-matching and the fully-decoupled SOI/SOG (silicon on glass) gyroscope, which mainly focuses on the MEMS gyroscope-sensing element [2,3,4,5,6,7,8]. Previous published works mainly focus on the superb mechanical characteristics of the MEMS gyroscope sensing element, which has a high requirement for processing technology. In this work, only the bias drift self-calibration method which can improve the accuracy of MEMS gyroscope at a general processing level is concerned.

The bias drift of the traditional gyroscopes is mainly composed of quadrature error and in-phase error. Quadrature error with a 90° offset from Coriolis signal can be eliminated by quadrature closed-loop feedback technique. However, phase delay existing in the practical system can make the quadrature error coupled to the angular rate sense path. Furthermore, because the quadrature errors are usually much larger than the angular rate signal, the bias drift in the sensed output signal can be increased significantly. Many studies of the quadrature error self-calibration method have been executed. The method presented in [9] is dual ramp method which works continuously without “dead-zones” where no rate is measured, but the system bandwidth and noise performance are degraded due to interrupted sampling and reduced average resonator amplitude. In [10], an improved design for the dual damp method which has good short-term noise characteristics has been proposed. The work in [11] implemented a background calibration method for MEMS gyroscopes which can calibrate both bias drift and scale factor simultaneously. The quadrature errors’ leak into the rate output signal is suppressed by monitoring the phase relationship between velocity and force. However, the self-calibration methods for MEMS gyroscopes in previous works mainly focus on the reduction of quadrature errors; hence, the methods of in-phase errors suppression were not well researched.

The mode reversal method utilizes the equality of coupling terms from each mode to the other, to eliminate the bias drift from the gyroscope output, which is an effective self-calibration method for both quadrature and in-phase errors’ cancelling. Rozelle reported the first mode reversal method for the hemispherical resonator gyroscope in 2010 [12]. However, it only can be used on the gyroscope which is geometrically symmetrical about its axis and results in “dead-zones” where no rate is measured. Kline reported a continuous-time mode reversal for bias drift suppression of a MEMS pendular gyroscope, and the driven-resonator and sensed-resonator modes are gradually rotated instead of abruptly switching, which avoids the “dead-zones” of operation [13]. The driven-resonator and sensed-resonator modes are gradually rotated instead of abruptly switching the drive and sense mode. However, the method requires a complex controller and a complicated demodulation algorithm which makes the system be more sensitive to the errors. In previously published work, these mode reversal methods for MEMS gyroscope periodically switched the resonator of drive and sensing mode to obtain an estimate of angular rate without bias drift, just like correlated double sampling in circuits. However, the periodic switching between the operation modes limits the operational bandwidth of gyroscope system and the correlate double sample (CDS) operation causes additional noise folding [14]. 

In this paper, we propose a bias drift self-calibration method for MEMS gyroscopes based on noise-suppressed mode reversal, and this method does not require modeling of the bias drift signal. The proposed self-calibration method: (1) uses mode reversal method and continuously samples the rate output to extract the measurement signal; (2) uses a linear combination algorithm to obtain an estimate of bias error and the compensated rate output; (3) decomposes the rate output signal by an improved complete ensemble empirical mode decomposition with adaptive noise (CEEMDAN); and (4) utilizes threshold denoising algorithm to filter the decomposition results and reconstructs them to obtain the desired rate outputs. The proposed method is advantageous over the previous method in that: (1) it has a relatively simple control system to ensure an acceptable bandwidth and can work without modeling of bias drift signal; (2) the noise performance of rate outputs has been significantly improved by utilizing the threshold denoising based on an improved CEEMDAN.

The remaining parts of this paper are structured as follows. The self-calibration method based on noise-suppressed mode reversal is shown in Section 2, including the principle and theoretical analysis of mode reversal, the threshold denoising algorithm based on the improved-CEEMDAN, and the proposed self-calibration system. The implementation of the proposed self-calibration system and the relevant experimental testing is given in Section 3; finally, Section 4 concludes the whole paper. 

## 2. The Self-Calibration Method Based on Noise-Suppressed Mode Reversal

### 2.1. The Principle and Theoretical Analysis of Mode Reversal

A simplified dynamic model of the MEMS gyroscope having two degrees of freedom can be described as:

(1) $$\{\begin{array}{c} m\ddot{x}+d_{1}\dot{x}+k_{1}x\;=\tau_{x}+2m\Omega_{z}\dot{y}\\ m\ddot{y}+d_{2}\dot{y}+k_{2}y=\tau_{y}-2m\Omega_{z}\dot{x} \end{array}.$$

As shown in Equation (1), only the component of the input angular rate along the z-axis causes a coupling between the x-axis and y-axis in an ideal gyroscope. However, manufacture imperfections always exist, which lead to an anisotropic and asymmetric gyroscope structure, resulting in dynamic coupling between the x-axis and y-axis. The asymmetry and anisoelasticity of the structure can be captured as misalignment of principal axis of spring from the physical axis of gyroscope structure. In addition to the structural asymmetry, misalignment of the actuators also contribute to the asymmetric spring terms.

**Figure 1:**
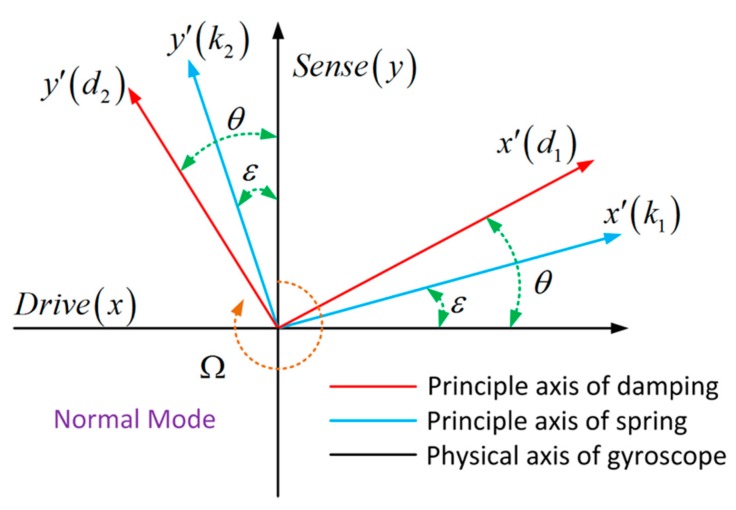
Schematic diagram of spring and damping constant misalignment caused by manufacture imperfections in normal operation mode.

Assume that the principal spring constants are $k_{1}$ and $k_{2}$, and the principal spring axes are tilted by angle ε with respect to the drive and sense axis—caused by fabrication imperfections, as shown in Figure 1. 

Let {*x, y*} be the physical drive and sense axis, and {*x′, y′*} be the principal axis of drive and sense resonator. Then, the relationship between spring forces and spring constants in the principal axes is given by:

(2) $$\left[\begin{array}{c} F_{x}^{\prime }\\ F_{y}^{\prime } \end{array}\right]=\left[\begin{array}{cc} k_{1} & 0\\ 0 & k_{2} \end{array}\right]\left[\begin{array}{c} x^{\prime }\\ y^{\prime } \end{array}\right].$$

Since the principal spring constants and physical axes are related by:

(3) $$\left[\begin{array}{c} x^{\prime }\\ y^{\prime } \end{array}\right]=\left[\begin{array}{cc} cos\varepsilon & -sin\varepsilon \\ sin\varepsilon & cos\varepsilon \end{array}\right]\left[\begin{array}{c} x\\ y \end{array}\right],$$

the spring constants in physical drive and sense axis are given by:

(4) $$\left[\begin{array}{c} F_{x}\\ F_{y} \end{array}\right]=\left[\begin{array}{cc} k_{xx} & k_{xy}\\ k_{yx} & k_{yy} \end{array}\right]\left[\begin{array}{c} x\\ y \end{array}\right].$$

As shown in Figure 1, the spring forces of principal axes and the actual spring force along the physical drive and sense axis are related by:

(5) $$\left[\begin{array}{c} F_{x}\\ F_{y} \end{array}\right]=\left[\begin{array}{cc} cos\varepsilon & sin\varepsilon \\ -sin\varepsilon & cos\varepsilon \end{array}\right]\left[\begin{array}{c} F_{x}^{\prime }\\ F_{y}^{\prime } \end{array}\right].$$

From Equations (2)–(5), we obtain:

(6) $$\left[\begin{array}{c} F_{x}\\ F_{y} \end{array}\right]=\left[\begin{array}{cc} cos\varepsilon & sin\varepsilon \\ -sin\varepsilon & cos\varepsilon \end{array}\right]\left[\begin{array}{c} F_{x}^{\prime }\\ F_{y}^{\prime } \end{array}\right]=\left[\begin{array}{cc} cos\varepsilon & sin\varepsilon \\ -sin\varepsilon & cos\varepsilon \end{array}\right]\left[\begin{array}{cc} k_{1} & 0\\ 0 & k_{2} \end{array}\right]\left[\begin{array}{c} x^{\prime }\\ y^{\prime } \end{array}\right]=\left[\begin{array}{cc} cos\varepsilon & sin\varepsilon \\ -sin\varepsilon & cos\varepsilon \end{array}\right]\left[\begin{array}{cc} k_{1} & 0\\ 0 & k_{2} \end{array}\right]\left[\begin{array}{cc} cos\varepsilon & -sin\varepsilon \\ sin\varepsilon & cos\varepsilon \end{array}\right]\left[\begin{array}{c} x\\ y \end{array}\right],$$

since the matrix of the actual spring constant can be represented as:

(7) $$\left[\begin{array}{cc} k_{xx} & k_{xy}\\ k_{yx} & k_{yy} \end{array}\right]=\left[\begin{array}{cc} cos\varepsilon & sin\varepsilon \\ -sin\varepsilon & cos\varepsilon \end{array}\right]\left[\begin{array}{cc} k_{1} & 0\\ 0 & k_{2} \end{array}\right]\left[\begin{array}{cc} cos\varepsilon & -sin\varepsilon \\ sin\varepsilon & cos\varepsilon \end{array}\right],$$

where

(8) $$k_{xx}=k_{1}cos^{2}\varepsilon +k_{2}sin^{2}\varepsilon =\frac{k_{1}+k_{2}}{2}+\frac{k_{1}-k_{2}}{2}cos2\varepsilon$$

(9) $$k_{xy}=k_{yx}=\frac{k_{1}-k_{2}}{2}sin2\varepsilon$$

(10) $$\;k_{yy}=k_{1}sin^{2}\varepsilon +k_{2}cos^{2}\varepsilon =\frac{k_{1}+k_{2}}{2}+\frac{k_{2}-k_{1}}{2}cos2\varepsilon .$$

Asymmetric damping can also be represented in terms of principal axes. The coordinate of the principal axis and the magnitude of the damping are determined by an averaging of the damping asymmetries. Similarly to the spring stiffness coefficients case, if damping constants along the principal axes are given by $d_{1}$ and $d_{2}$, and the axes are tilted by an angle θ from physical axes of gyroscope, then the damping constant coefficients in physical axes are shown as:

(11) $$d_{xx}=d_{1}cos^{2}\theta +d_{2}sin^{2}\theta =\frac{d_{1}+d_{2}}{2}+\frac{d_{1}-d_{2}}{2}cos2\theta$$

(12) $$d_{xy}=d_{yx}=\frac{d_{1}-d_{2}}{2}sin2\theta$$

(13) $$d_{yy}=d_{1}cos^{2}\theta +d_{2}sin^{2}\theta =\frac{d_{1}+d_{2}}{2}+\frac{d_{2}-d_{1}}{2}cos2\theta .$$

Taking into account fabrication imperfections, the dynamic equation, Equation (1), is modified as follows:

(14) $$\{\begin{array}{c} m\ddot{x}+d_{xx}\dot{x}+d_{xy}\dot{y}+k_{xx}x+k_{xy}y\;=\tau_{x}+2m\Omega \dot{y}\\ m\ddot{y}+d_{yx}\dot{x}+d_{yy}\dot{y}+k_{yx}x+k_{yy}y=\tau_{y}-2m\Omega \dot{x} \end{array}.$$

Equation (14) is the governing equation for the gyroscope in the normal operation mode. Assume that drive displacement $x=q_{0}\cos\left(\omega_{x}t\right)$, where the $q_{0}$ is the magnitude of the drive displacement and $\omega_{x}$ is the natural frequency of drive mode (x-axis). By substituting the stable state condition of feedback loop and the drive displacement into Equation (14), we obtain the sense force $\tau_{y}$ as:

(15) $$\tau_{y}=k_{yx}q_{0}cos\omega_{x}t-q_{0}\omega_{x}\left(d_{yx}+2m\Omega \right)sin\omega_{x}t.$$

Through the interface module, demodulation module and low-pass filter, the output signal which contains the angular rate is represented as:

(16) S=SF⋅Ω+Bias ,

where

(17) $$\{\begin{array}{c} SF=\frac{1}{mq_{0}\sqrt{\frac{\left(k_{1}+k_{2}\right)+\left(k_{1}-k_{2}\right)cos2\varepsilon }{2m}}cos\varphi }\\ Bias=\frac{1}{2}q_{0}\left[\left(\frac{k_{2}-k_{1}}{2}\right)sin2\varepsilon \cdot sin\varphi -\left(\frac{d_{2}-d_{1}}{2}\right)sin2\theta \cdot cos\varphi \sqrt{\frac{\left(k_{1}+k_{2}\right)+\left(k_{1}-k_{2}\right)cos2\varepsilon }{2m}}\right] \end{array}$$

and φ denotes the total phase delay of the interface module, demodulation module, and low-pass filter. SF and Bias denote the scale factor and bias of the gyroscope in normal operation mode respectively.

**Figure 2:**
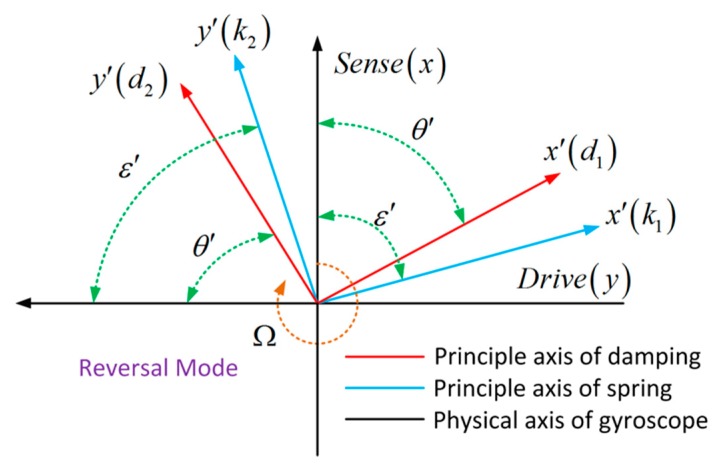
Schematic diagram of spring and damping constant misalignment caused by manufacture imperfections in reverse operation mode.

When the gyroscope in the reverse operation mode, the drive resonator and sense resonator are switched. The location of principal axes of drive and sense mode remains the same, and the coordinates of physical axes rotate 90° which means that $\varepsilon^{\prime }=\frac{\pi }{2}-\varepsilon$ and $\theta^{\prime }=\frac{\pi }{2}-\theta$, as shown in Figure 2.

Since Equation (3) and Equation (5) are changed to:

(18) $$\left[\begin{array}{c} x^{\prime }\\ y^{\prime } \end{array}\right]=\left[\begin{array}{cc} -sin\varepsilon & -cos\varepsilon \\ cos\varepsilon & -sin\varepsilon \end{array}\right]\left[\begin{array}{c} x\\ y \end{array}\right]$$

(19) $$\left[\begin{array}{c} F_{x}\\ F_{y} \end{array}\right]=\left[\begin{array}{cc} -sin\varepsilon & -cos\varepsilon \\ -cos\varepsilon & -sin\varepsilon \end{array}\right]\left[\begin{array}{c} F_{x}^{\prime }\\ F_{y}^{\prime } \end{array}\right],$$

we obtained the the actual spring constants matrix of mode reversal as follows:

(20) $$\left[\begin{array}{cc} k_{xx}^{\prime } & k_{xy}^{\prime }\\ k_{yx}^{\prime } & k_{yy}^{\prime } \end{array}\right]=\left[\begin{array}{cc} -sin\varepsilon & -cos\varepsilon \\ -cos\varepsilon & -sin\varepsilon \end{array}\right]\left[\begin{array}{cc} k_{1} & 0\\ 0 & k_{2} \end{array}\right]\left[\begin{array}{cc} -sin\varepsilon & -cos\varepsilon \\ cos\varepsilon & -sin\varepsilon \end{array}\right],$$

where

(21) $$k_{xx}^{\prime }=k_{1}cos^{2}\varepsilon +k_{2}sin^{2}\varepsilon =\frac{k_{1}+k_{2}}{2}+\frac{k_{2}-k_{1}}{2}cos2\varepsilon$$

(22) $$k_{xy}^{\prime }=k_{yx}^{\prime }=\frac{k_{1}-k_{2}}{2}sin2\varepsilon$$

(23) $$k_{yy}^{\prime }=k_{1}sin^{2}\varepsilon +k_{2}cos^{2}\varepsilon =\frac{k_{1}+k_{2}}{2}+\frac{k_{1}-k_{2}}{2}cos2\varepsilon .$$

Similarly, the damping constant coefficients in physical axes are shown as:

(24) $$d_{xx}^{\prime }=d_{1}cos^{2}\theta +d_{2}sin^{2}\theta =\frac{d_{1}+d_{2}}{2}+\frac{d_{2}-d_{1}}{2}cos2\theta$$

(25) $$d_{xy}^{\prime }=d_{yx}^{\prime }=\frac{d_{1}-d_{2}}{2}sin2\theta$$

(26) $$d_{yy}^{\prime }=d_{1}cos^{2}\theta +d_{2}sin^{2}\theta =\frac{d_{1}+d_{2}}{2}+\frac{d_{1}-d_{2}}{2}cos2\theta .$$

Therefore, the governing equation for the gyroscope in the mode reversal operation mode can be described as:

(27) $$\{\begin{array}{c} m\ddot{x}+d_{xx}^{\prime }\dot{x}+d_{xy}^{\prime }\dot{y}+k_{xx}^{\prime }x+k_{xy}^{\prime }y\;=\tau_{x}^{\prime }+2m\Omega \dot{y}\\ m\ddot{y}+d_{yy}^{\prime }\dot{\dot{y}+d_{yx}^{\prime }\dot{x}}+k_{yx}^{\prime }x+k_{yy}^{\prime }y=\tau_{y}^{\prime }-2m\Omega \dot{x} \end{array}.$$

Assume that drive displacement $y=q_{0}sin\left(\omega_{y}^{\prime }t\right)$, where the $q_{0}$ is the magnitude of the drive displacement and $\omega_{y}^{\prime }$ is the natural frequency of drive mode (y-axis). By substituting the stable state condition of feedback loop and the drive displacement into Equation (20), we obtain the sense force $\tau_{x}^{\prime }$ as:

(28) $$\tau_{x}^{\prime }=k_{xy}^{\prime }q_{0}sin\omega_{y}^{\prime }t+q_{0}\omega_{y}^{\prime }cos\left(\omega_{y}^{\prime }t\right)\left(d_{xy}^{\prime }-2\Omega m\right).$$

Through the signal processing module includes that interface circuit, demodulation circuit, and low-pass filter, the output signal which contains the angular rate is written as:

(29) $$S^{\prime }=SF^{\prime }\cdot \Omega +{\mathrm{Bias}}^{\prime },$$

where

(30) $$\{\begin{array}{c} SF^{\prime }=\frac{1}{mq_{0}\sqrt{\frac{\left(k_{1}+k_{2}\right)+\left(k_{1}-k_{2}\right)cos2\varepsilon }{2m}}cos\varphi }\\ Bias^{\prime }=-\frac{1}{2}q_{0}\left[\left(\frac{k_{2}-k_{1}}{2}\right)sin2\varepsilon \cdot sin\varphi -\left(\frac{d_{2}-d_{1}}{2}\right)sin2\theta \cdot cos\varphi \sqrt{\frac{\left(k_{1}+k_{2}\right)+\left(k_{1}-k_{2}\right)cos2\varepsilon }{2m}}\right] \end{array}$$

and φ denotes the phase delay of the signal processing module which has a value identical to normal in operation mode. $SF^{\prime }$ and $Bias^{\prime }$ denote the scale factor and bias of the gyroscope in mode reversal operation mode respectively.

From Equations (17) and (30), the estimation of bias drift and measured angular rate can be obtained as:

(31) $$SF=SF^{\prime }$$

(32) $$Bias=-Bias^{\prime }=\left(S-S^{\prime }\right)/2$$

(33) $$\Omega =\left(S+S^{\prime }\right)/2SF.$$

As shown in Equation (33), the bias drift can be eliminated by utilizing the two rate outputs of normal operation mode and reverse operation mode, and double the sensitivity of angle rate.

However, the typical mode reversal scheme degrades the overall noise performance by folding uncorrelated noise from the upper bands down to the baseband and reduced measurement bandwidth, which is limited, just like correlated double sampling method in circuits. Hence, the threshold denoising method was adopted to enhance the noise performance, and an additional gyroscope was applied to increase the bandwidth.

### 2.2. The Threshold Denoising Algorithm Based on Improved CEEMDAN

Empirical mode decomposition (EMD) is an adaptive analysis method for non-stationary signals in nonlinear systems [15]. A series of functions which are called intrinsic mode functions (IMFs) can be obtained by the decomposition of original signal using EMD. However, the original algorithm of EMD exists as an un-desirable phenomenon which is called “mode mixing.” In order to solve this issue, the ensemble empirical mode decomposition (EEMD) was presented in [16]. The EEMD utilized the additional white Gaussian noise to suppress the mode mixing by making use of the dyadic filter bank behavior. But EEMD also created new difficulties such that a lot of residual noise was introduced in the reconstructed signal, which is the sum of the modes and the final trend. The Complementary EEMD, which is presented in [17], can reduce the residual noise in signal reconstruction by using complementary pairs of noise expressively. Nevertheless, the complementary pairs of noise may generate a large number of additional modes. 

**Figure 3:**
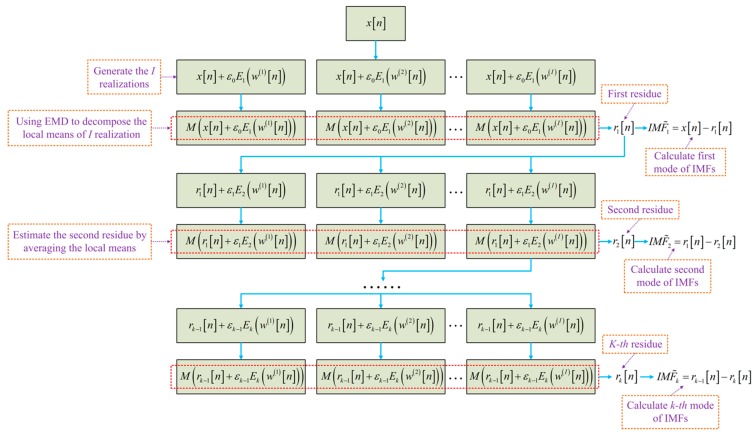
Flowchart describing the improved version of complete ensemble empirical mode decomposition with adaptive noise (CEEMDAN).

The complete ensemble empirical mode decomposition with adaptive noise (CEEMDAN) achieves a negligible residual noise, and the problem of additional modes caused by complementary pairs of noise has also been solved. However, CEEMDAN still has some problems to be solved: there are some residual noise in the modes and spurious modes occur in the early phase of decomposition. In order to avoid the problems, in previous works, the improved CEEMDAN algorithm [18] was adopted. The flowchart of this algorithm is shown as Figure 3. In Figure 3, $E_{k}\left(\cdot \right)$ is the operator of the *k*-th mode of EMD extraction, M(·) is the operator of local mean, $w^{\left(i\right)}$ is the Gaussian white noise with zero mean unit variance, and the constant $\beta_{k}$ is set as $\beta_{k}=\varepsilon_{k}std\left(r_{k}\right)$. 

The set of $IMF_{1},IMF_{2},\ldots ,IMF_{k}$ is the final result of the decomposition of the original signal. 

A set of IMFs is obtained by the decomposition of the original signal using improved CEEMDAN method. Both useful components and color noise are contained in the set of IMFs; thus, the threshold denoising method should be adopted [19]. The $imf_{k}$ which is the points in the *k*-th IMF, can be expressed as $\left\{d_{k}^{\left(j\right)},j=1,\ldots ,N\right\}$, and the estimation of empirical variance of $imf_{k}$ is shown in Equation (34):

(34) $$\hat{V}\left(imf_{k}\right)=\frac{1}{N}\sum_{j=1}^{N}{\left(d_{k}^{\left(j\right)}\right)}^{2}.$$

The information of desired and noise signal is all contained in $\hat{V}\left(imf_{k}\right)$, and the variance of pure noise should be calculated, which is to determine the threshold of denoising. Therefore, the sample entropy (SE) has been adopted [20]. The SE value of signal is positively correlated with the amount of noise in signal. SE is primarily determined by tow parameters: the embedding dimension *m* and the tolerance value *r*. The formula for calculating SE can be shown as follows:

(35) $$SE\left(m,r\right)=\underset{N\to \infty }{lim}\left\{-ln\left[\frac{A^{m}r}{B^{m}r}\right]\right\}.$$

After the calculation of sample entropy of each IMF, the first two IMFs which have the highest sample entropy are selected as pure noise signal. The robust estimator shown in Equation (36) can be applied to estimate the standard deviations of noise contained in the two selected IMFs:

(36) $$\hat{\sigma }\left(k\right)=\frac{median\left(\left|d_{k}^{\left(j\right)},j=1,\ldots ,N\right|\right)}{0.6754},k=1,2.$$

The noise of MEMS gyroscope can be modeled by fractional Gaussian noise; therefore, the variances of the noise that contained in IMFs can be calculated by Equations (36) and (37). Similarly to the wavelet threshold denoising, the threshold of the denoising algorithm based on improved CEEMDAN can be obtained by Equation (38).

(37) $$\hat{V}\left(k\right)=\rho_{H}^{\left(2H-2\right)\left(k^{\prime }-k\right)}\hat{V}\left(2\right)=\rho_{H}^{\left(2H-2\right)\left(k^{\prime }-k\right)}{\left[\hat{\sigma }\left(2\right)\right]}^{2},k>2$$

(38) $$\hat{T_{k}}=\sqrt{\hat{V}\left(k\right)*2lnN}=\hat{\sigma }\left(k\right)\sqrt{2lnN}.$$

The commonly used threshold denoising algorithm mainly includes two types: the hard-threshold and soft-threshold. The hard-threshold denoising is shown as below:

(39) $${\tilde{h}}^{\left(i\right)}\left(t\right)=\{\begin{array}{cc} h^{\left(i\right)}\left(t\right), & \left|h^{\left(i\right)}\left(t\right)\right|>T_{i}\\ 0, & \left|h^{\left(i\right)}\left(t\right)\right|\leq T_{i} \end{array}.$$

Similarly, the soft-threshold denoising can be expressed as:

(40) $${\tilde{h}}^{\left(i\right)}\left(t\right)=\{\begin{array}{cc} sgn\left(h^{\left(i\right)}\left(t\right)\right)\left(\left|h^{\left(i\right)}\left(t\right)\right|-T_{i}\right), & \left|h^{\left(i\right)}\left(t\right)\right|>T_{i}\\ 0, & \left|h^{\left(i\right)}\left(t\right)\right|\leq T_{i} \end{array}.$$

Since it can make the de-noised waveform smoother, the soft-threshold denoising is adopted in this paper.

### 2.3. The Self-Calibration System Based on Noise-Suppressed Mode Reversal

**Figure 4:**
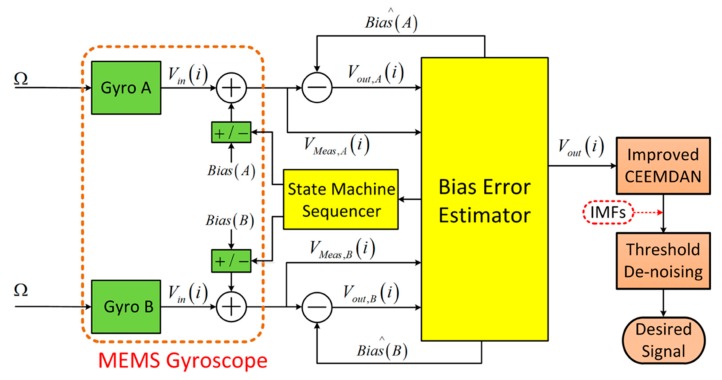
Block diagram of the two collinear gyroscope, self-calibrating system based on noise-suppressed mode reversal.

The single gyroscope bias drift self-calibration model described in Section 2.1 is helpful for principle analysis and performance projections; nevertheless, several difficulties have to be solved to realize a practical real-time self-calibrated system with uninterrupted output. Such a system can be designed using an additional gyroscope for a single axis system. The single axis system in this work consists of two collinear gyroscopes, a state machine sequencer, a bias estimator, and the threshold denoising algorithm based on improved CEEMDAN; it is shown in Figure 4. 

**Figure 5:**
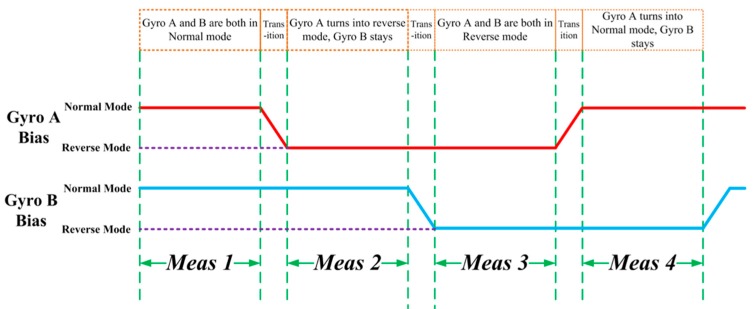
Illustration of the state machine sequencer which enables full observability of angle rate and gyroscope bias drifts.

The state machine is illustrated in Figure 5. As shown in Figure 5, bias error signal polarities are associated with gyroscopes A and B respectively. According to the analysis in the Section 2.1, the detected input rate signal has a positive relationship with the bias error signal when the gyroscope is in normal operation mode, and a negative relationship when the gyroscope is in reverse operation mode.

The graph shows four different measurement intervals, Meas1 to Meas4. The operation mode of the gyroscopes A and B switch alternately between normal and reverse mode according to the sequence of state, which enables uninterrupted real-time bias drift calibration. The respective bias error signals of the gyroscopes Bias(A) and Bias(B) also switch between the positive and negative polarities, and make bias drifts of both gyroscopes observable by the self-calibration bias estimator.

The measurement signal $V_{Meas,A}$ consists of detecting angular rate signal $V_{in}$ and bias error signal of Gyro A Bias(A). Similarly, measurement signal $V_{Meas,B}$ consists of detecting angular rate signal $V_{in}$ and bias error signal of Gyro B Bias(B). The bias estimator receives the measurement signals $V_{Meas,A}$ and $V_{Meas,B}$ and the calibrated outputs $V_{out,A}$ and $V_{out,B}$ which are used to estimate bias error in next measurement interval. The estimated bias error signal $\hat{Bias}\left(A\right)$ and $\hat{Bias}\left(B\right)$ are generated by the bias estimator, which are used for error calibration to obtain more accurate measurement.

Both gyroscopes are in a stable operational state during each measurement interval, and the sense axes of the input angle rates are identical. The measurement signals of Gyro A and Gyro B in the *i*-th measurement interval MeasA(i) and MeasB(i) are shown below, respectively:

(41) MeasA(i)=Ω(i)⋅SF+/−Bias(A)

(42) MeasB(i)=Ω(i)⋅SF+/−Bias(B)

For the first and second intervals:

(43) MeasA(1)=Ω(1)⋅SF+Bias(A)

(44) MeasB(1)=Ω(1)⋅SF−Bias(B)

(45) MeasA(2)=Ω(2)⋅SF−Bias(A)

(46) MeasB(2)=Ω(2)⋅SF+Bias(B).

The above Equations (43)–(46) are transformed into a matrix representation:

(47) $$\left[\begin{array}{c} \begin{array}{c} MeasA\left(1\right)\\ MeasB\left(1\right)\\ MeasA\left(2\right) \end{array}\\ MeasB\left(2\right) \end{array}\right]=H\left[\begin{array}{c} \Omega \left(1\right)\cdot SF\\ \Omega \left(2\right)\cdot SF\\ Bias\left(A\right)\\ Bias\left(B\right) \end{array}\right]=\left[\begin{array}{c} V_{in}\left(1\right)\\ V_{in}\left(2\right)\\ Bias\left(A\right)\\ Bias\left(B\right) \end{array}\right],$$

where H is shown as:

(48) $$H=\left[\begin{array}{c} 1\\ 1\\ 0\\ 0 \end{array}\;\;\;\begin{array}{c} 0\\ 0\\ 1\\ 1 \end{array}\;\;\;\begin{array}{c} 1\\ 0\\ -1\\ 0 \end{array}\;\;\;\begin{array}{c} 0\\ 1\\ 0\\ 1 \end{array}\right].$$

The inverse matrix of matrix H is given in Equation (49):

(49) $$H^{-1}=\left[\begin{array}{c} 0.5\\ 0.5\\ 0.5\\ -0.5 \end{array}\;\;\;\begin{array}{c} 0.5\\ -0.5\\ -0.5\\ 0.5 \end{array}\;\;\;\begin{array}{c} 0.5\\ 0.5\\ -0.5\\ -0.5 \end{array}\;\;\;\begin{array}{c} -0.5\\ 0.5\\ 0.5\\ 0.5 \end{array}\right].$$

The four variables $V_{out}\left(1\right)$, $V_{out}\left(2\right)$, $\hat{Bias\left(A\right)}$, and $\hat{Bias\left(B\right)}$ are, therefore, individually observable, and can be obtained as:

(50) $$\left[\begin{array}{c} V_{out}\left(1\right)\\ V_{out}\left(2\right)\\ \hat{Bias\left(A\right)}\\ \hat{Bias(B}) \end{array}\right]=\left[\begin{array}{c} 0.5\\ 0.5\\ 0.5\\ -0.5 \end{array}\;\;\;\begin{array}{c} 0.5\\ -0.5\\ -0.5\\ 0.5 \end{array}\;\;\;\begin{array}{c} 0.5\\ 0.5\\ -0.5\\ -0.5 \end{array}\;\;\;\begin{array}{c} -0.5\\ 0.5\\ 0.5\\ 0.5 \end{array}\right]\left[\begin{array}{c} \begin{array}{c} MeasA\left(1\right)\\ MeasB\left(1\right)\\ MeasA\left(2\right) \end{array}\\ MeasB\left(2\right) \end{array}\right].$$

The final output signal of the bias estimator $V_{out}$ is calibrated output signal, which can be obtained by Equation (50). 

After the calibration by mode reversal, the threshold denoising is used to eliminate the noise in the calibrated signal. The process of threshold denoising algorithm is shown in Figure 4. A set of IMFs is obtained by the decomposition of the calibrated signal using improved CEEMDAN method. Then, we can determine the IMF with dominated noise by calculating the sample entropy of each IMFs, and substitute the variance of noise dominant IMF into the factional Gaussian noise model given in Equation (38) to obtain the threshold of denoising algorithm. Finally, the desired signal can be obtained by reconstruction of the de-noised IMFs.

## 3. Experimental Results and Discussion

### 3.1. The Implemention of the Proposed Self-Calibration System

**Figure 6:**
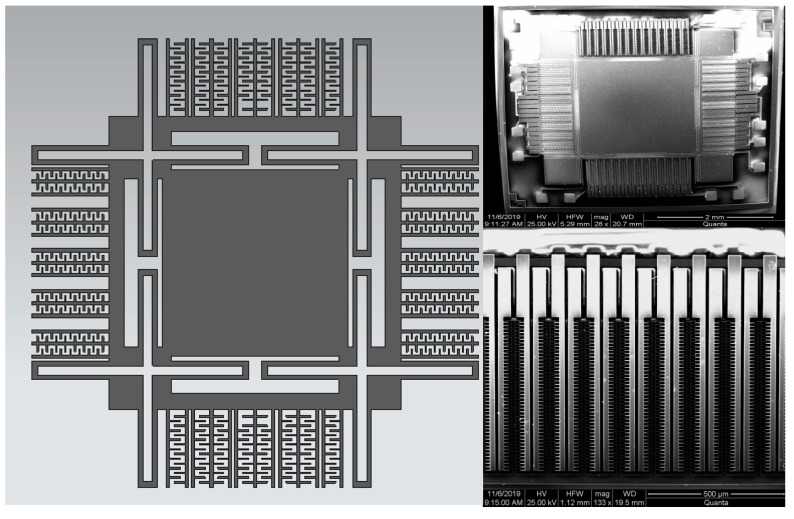
The diagram of the micro-electromechanical systems (MEMS) gyroscope and a SEM of the sensing element of the gyroscope.

To verify the feasibility of the proposed self-calibration system, the relevant experiment was carried out. The gyroscope we tested which has a symmetrical structure is fabricated by SOI processing, as shown in Figure 6, and the total dimensions of the sensing element are 4 mm× 4 mm [21]. 

The key parameters of the symmetric gyroscope are shown in Table 1. 

**Figure 7:**
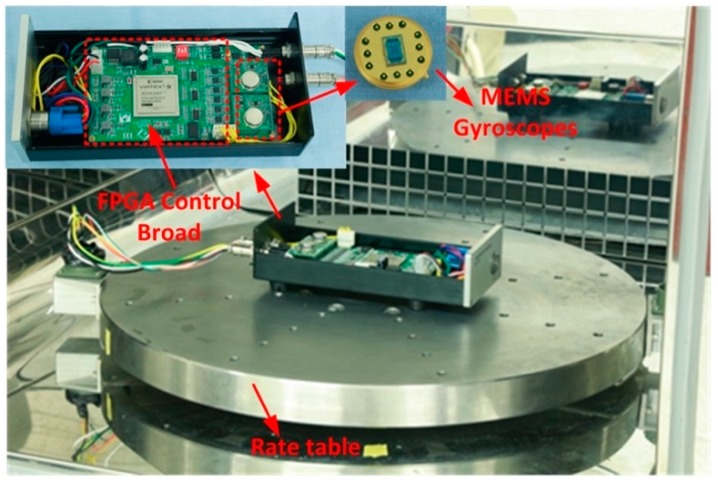
Control circuit for the packaged gyroscopes is mounted on rate table in an oven chamber.

The two packaged gyroscopes with identical scale factors were arranged on two, identical four-layer print circuit boards (PCB), and the rest of electronic components were mounted on a 100 mm× 80 mm, six-layer PCB, as shown in Figure 7.

The two identical PCBs connected the structures of the weak signals. The third PCB was composed of the 24-bit resolution analog-to-digital converters ADS131 (Texas Instruments Company, Dallas, TX, USA); a FPGA chip XC5VL (Xilinx Company, San Jose, CA, USA) in which the control loops, the demodulation module, state machine sequencer, bias error estimator, and threshold denoising algorithm were implemented; and the 16-bit resolution digital-to-analog converters AD5754 (Analog Device, Norwood, MA, USA).

### 3.2. Experimental Testing

**Figure 8:**
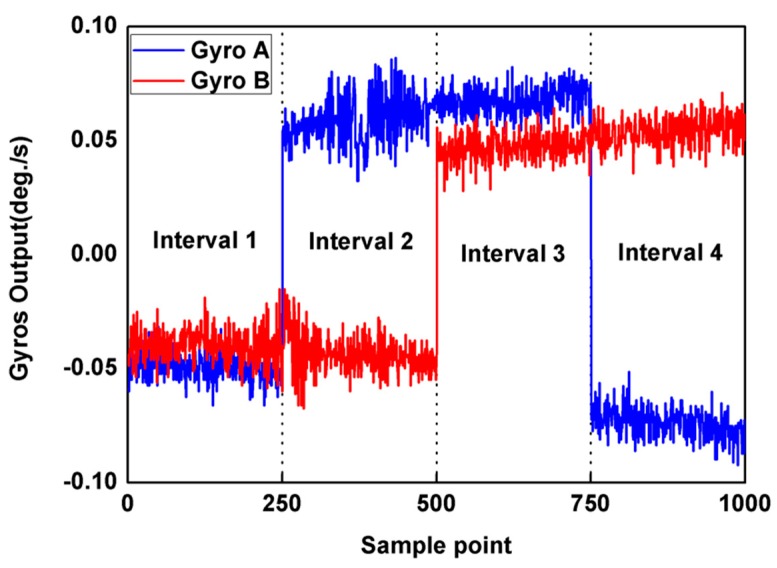
The output of gyroscopes operated with state machine sequencer.

The output signal data was collected with 50 Hz sampling frequency and was recorded in PC with 1 s average value. The outputs of two collinear gyroscopes were recorded for 1000 s at 25 °C controlled temperature, and each measurement interval contains 150 points, as shown in Figure 8. 

**Figure 9:**
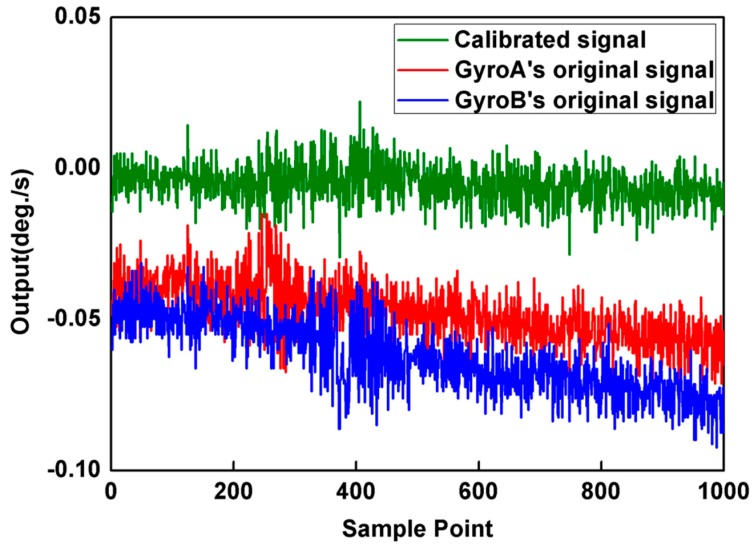
Time domain illustration of dual gyroscopes self-calibration.

In this figure, the normal mode portion is opposite to the reversal mode portion. Therefore, the bias drift error can be estimated and eliminated according to Equation (50). Detecting the outputs during equal time intervals of normal and reversal mode, the calibrated and un-calibrated responses in time domain are shown in Figure 9, respectively. 

**Figure 10:**
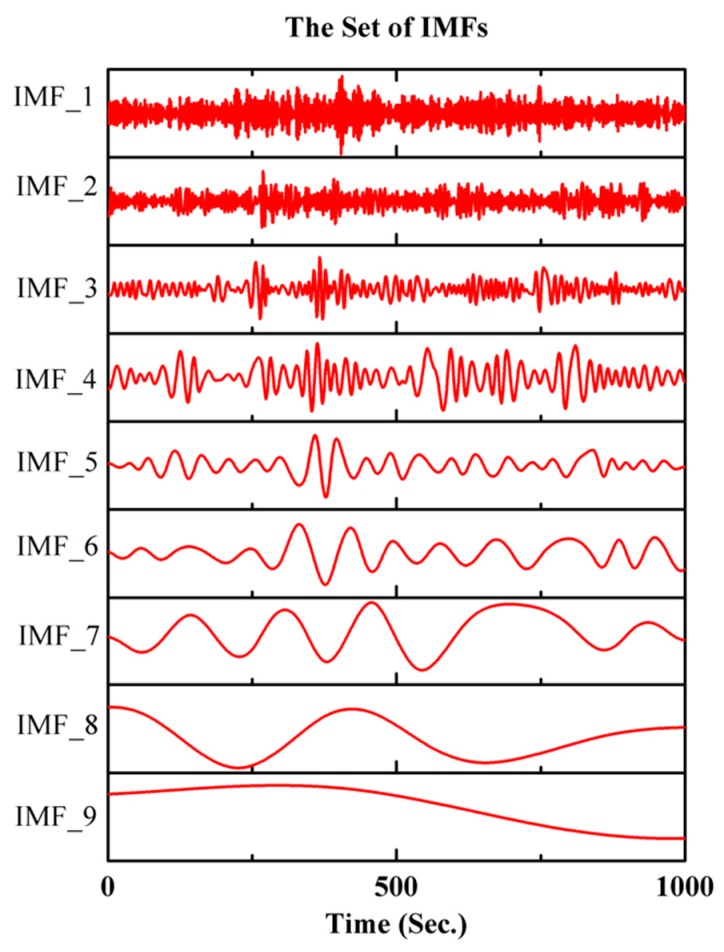
The decomposition results of the improved CEEMDAN algorithm.

From Figure 9, it is obvious that there is a large amount of irrelevant noise in the calibrated signal of mode reversal. In order to eliminate the influence of the additional noise in the calibrated signal on the measurement accuracy of MEMS gyroscopes, the threshold denoising method was adopted here. As described in Section 3, the first step of threshold denoising is to decompose the calibrated signal of mode reversal into a set of IMFs using the improved CEEMDAN. The decomposition results of calibrated signal of mode reversal are given in Figure 10. 

**Figure 11:**
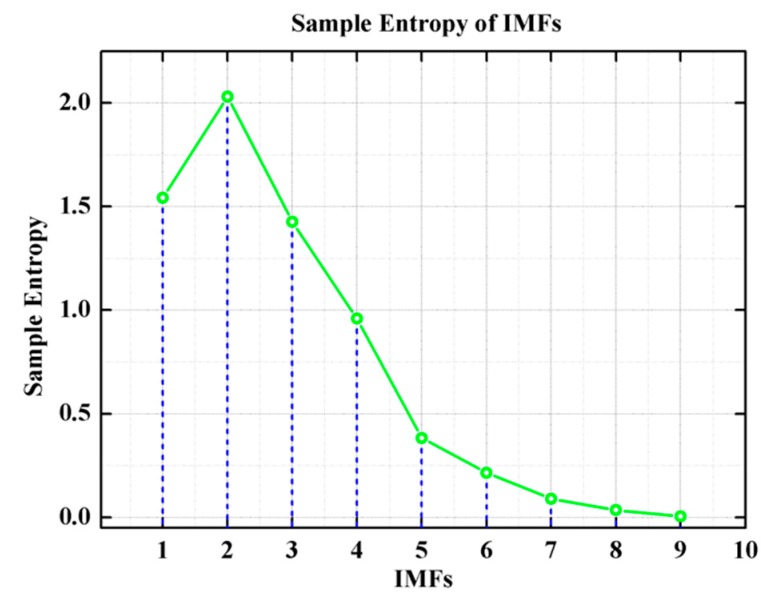
The sample entropy of the set of intrinsic mode functions (IMFs).

In order to find the noise dominant IMF from the set of IMFs, the sample entropy of each IMFs is calculated according to Equation (35). The calculated result of the sample entropy of each IMFs is shown in Figure 11.

From Figure 11, the IMF2 with local maximum value of sample entropy is selected as noise dominant component, and the variance of the IMF2 determines the denoising threshold of the filtering algorithm. 

**Figure 12:**
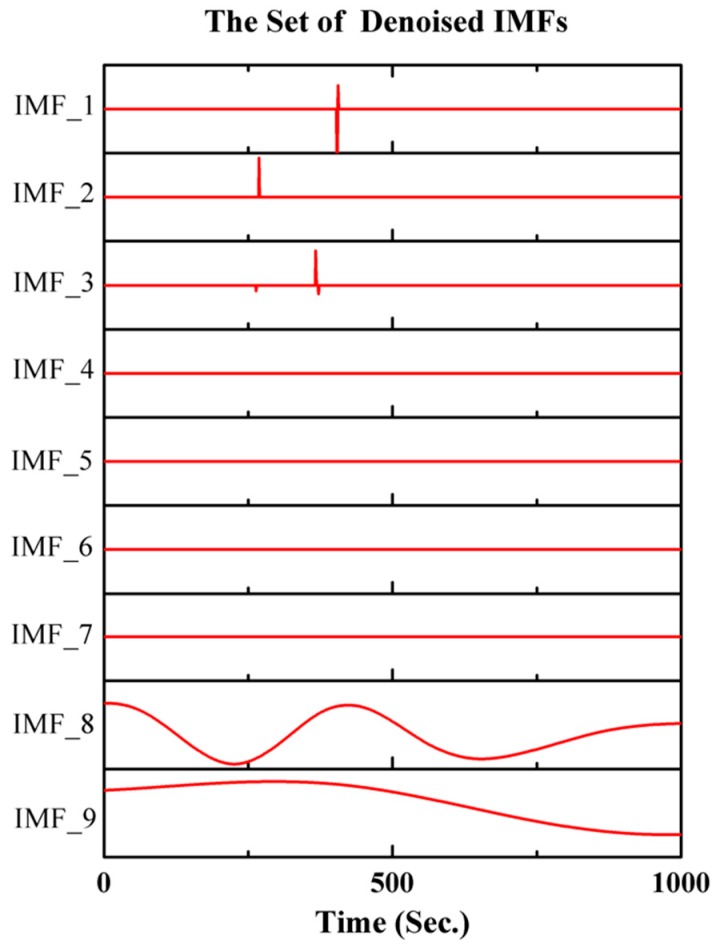
The filtered result of the IMFs after threshold denoising.

Figure 12 gives the filtered results of IMFs after threshold denoising. The de-noised calibrated signal is obtained by the reconstruction of the denoised IMFs. 

**Figure 13:**
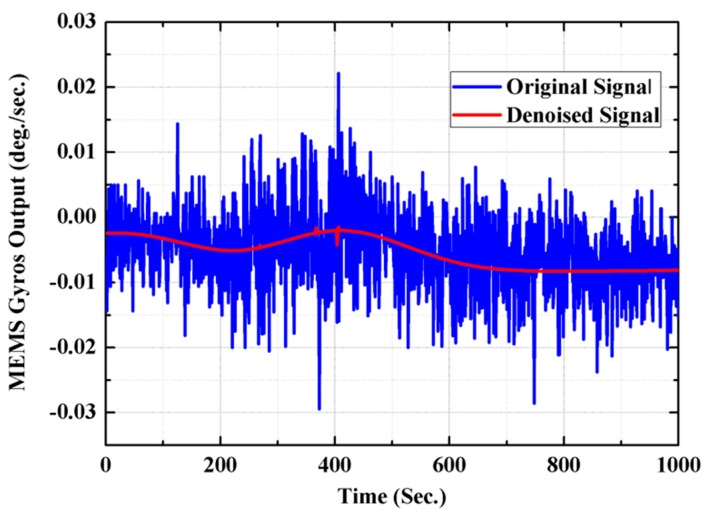
The comparison of original and denoised signal.

As shown in Figure 13, by comparing the calibrated signal before and after denoising, it can be concluded that the threshold denoising algorithm proposed in this paper can effectively filter out the noise in the calibrated signal.

**Figure 14:**
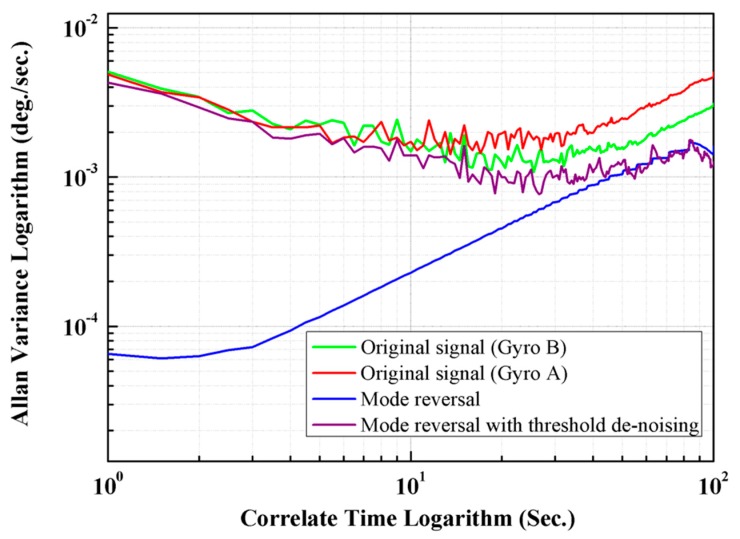
The Allan variance graphic of calibrated and un-calibrated gyroscopes’ outputs.

The Allan variance method, which is a widely used quantitative performance evaluation method for inertial sensors [22], was applied for the quantitative comparison of calibration results of different methods. Figure 14 shows the Allan variance analysis of un-calibrated output signals of Gyro A and B, and the calibration results of the typical mode reversal and the proposed improved CEEMDAN-mode reversal method; and Table 1 shows the quantitative results in detail. 

From Table 2, we can see that the proposed self-calibration method improves bias instability from 0.0066°/s and 0.0055°/s to 0.0011°/s, and the angle random walk decreases from 1.18 $\times 10^{-4}\;{}^{\circ}/s^{1/2}$ and 2.04 $\times 10^{-4}\;{}^{\circ}/s^{1/2}$ to 2.19 $\times 10^{-5}\;{}^{\circ}/s^{1/2}$.

Both Figure 14 and Table 2 show that compared with the typical method, the proposed mode reversal method with improved CEEMDAN can enhance the bias drift stability and the performance of noise suppression significantly.

## 4. Conclusions

In summary, a novel bias drift self-calibration method based on noise-suppressed mode reversal for MEMS gyroscopes is proposed. Different from other approaches, the proposed method does not require modeling of bias drift signal. Moreover, benefiting from the improved CEEMDAN threshold denoising, this method eliminates the the noise folding from upper bands down to baseband, which is caused by the mode reversal, and can improve the overall noise performance of the calibration system. The proposed mode reversal self-calibration method does not decrease system bandwidth by adding additional collinear gyroscope. Compared to the typical mode reversal method, the proposed method decreases the bias instability and angle random walk effectively, specifically in Allan variance coefficients, *B* from 0.0022°/s to 0.0011°/s and N from 5.67 $\times 10^{-4}\;{}^{\circ}/s^{1/2}$ to 2.19 $\times 10^{-5}\;{}^{\circ}/s^{1/2}$. The proposed mode reversal method may enable the MEMS gyroscopes to enter into tactical grade application where low-cost fiberoptic gyroscopes have traditionally dominated.

## Figures and Tables

**Table 1:** The key parameters of the gyroscope.

Parameter	Values
Drive mode resonant frequency	8195 Hz
Sense mode resonant frequency	8142 Hz
Quality factor of drive mode	184
Quality factor of sense mode	195
Drive effective mass	5.49$\;\times 10^{-7}$
Sense effective mass	5.3$\;\times 10^{-7}$
Capacitance of drive mode	4.5 pF
Capacitance of sense mode	4 pF

**Table 2:** Compensation result using different methods.

	Original (Gyro A)	Original (Gyro B)	Mode Reversal	Mode Reversal with Threshold Denoising
Q (μ rad)	0.0026	0.0021	0.0020	8.26$\;\times 10^{-4}$
$N\;\left({}^{\circ}/s^{1/2}\right)$	1.18$\;\times 10^{-4}$	2.04$\;\times 10^{-4}$	5.67$\;\times 10^{-4}$	2.19$\;\times 10^{-5}$
B (°/s)	0.0066	0.0055	0.0022	0.0011
$K\;\left({}^{\circ}/s^{3/2}\right)$	0.0456	0.0494	0.0513	0.0045
$R\;\left({}^{\circ}/s^{2}\right)$	0.2094	0.1709	0.302	0.0195
